# Accessory Interaction Motifs in the Atg19 Cargo Receptor Enable Strong Binding to the Clustered Ubiquitin-related Atg8 Protein*[Fn FN2]

**DOI:** 10.1074/jbc.M116.736892

**Published:** 2016-07-11

**Authors:** Christine Abert, Georg Kontaxis, Sascha Martens

**Affiliations:** From the Department of ‡ Biochemistry and Cell Biology, Max F. Perutz Laboratories, University of Vienna, Vienna Biocenter, Dr. Bohr-Gasse 9/3 and; the Department of § Structural and Computational Biology, Max F. Perutz Laboratories, University of Vienna, Campus Vienna Biocenter 5/1, 1030 Vienna, Austria

**Keywords:** autophagy, membrane, NMR, protein-protein interaction, ubiquitin, Atg8, autophagosome, cargo receptor, selective autophagy

## Abstract

Selective autophagy contributes to cellular homeostasis by delivering harmful material into the lysosomal system for degradation via vesicular intermediates referred to as autophagosomes. The cytoplasm-to-vacuole targeting pathway is a variant of selective autophagy in *Saccharomyces cerevisiae* during which hydrolases such as prApe1 are transported into the vacuole. In general, selectivity is achieved by autophagic cargo receptors that link the cargo to autophagosomal membranes because of their ability to simultaneously interact with the cargo and Atg8 proteins that coat the membrane. The Atg19 receptor contains multiple Atg8 interaction sites in its C terminus in addition to a canonical Atg8-interacting LC3-interacting region (LIR, with LC3 being a homolog of Atg8) motif, but their mode of interaction with Atg8 is unclear. Here we show, using a combination of NMR, microscopy-based interaction assays, and prApe1 processing experiments, that two additional sites interact with Atg8 in a LIR-like and thus mutually exclusive manner. We term these motifs accessory LIR motifs because their affinities are lower than that of the canonical LIR motif. Thus, one Atg19 molecule has the ability to interact with multiple Atg8 proteins simultaneously, resulting in a high-avidity interaction that may confer specific binding to the Atg8-coated autophagosomal membrane on which Atg8 is concentrated.

## Introduction

Autophagy is a homeostatic and cell-protective lysosomal degradation pathway in eukaryotic cells. Autophagy is triggered during lack of nutrients and serves to maintain basic cellular functions by transporting bulk cytoplasmic material into the lytic compartment for degradation in a rather non-selective manner. However, autophagy also has the ability to specifically target harmful components, such as damaged cell organelles, intracellular pathogens, or potentially toxic protein aggregates (1, 2).

Macroautophagy (referred to as autophagy hereafter) involves the *de novo* formation of a specialized double membrane organelle called the autophagosome. Autophagosomes originate from a membrane structure referred to as the isolation membrane or phagophore that gradually encloses cytoplasmic cargo material (3, 4). After their formation, autophagosomes fuse with lysosomes or the vacuole, wherein their inner membrane and the cargo material are degraded.

Selectivity of autophagy is conferred by cargo receptors that link the cargo to the isolation membrane because of their ability to simultaneously bind the cargo and the isolation membrane via ATG8 family proteins (1, 5). ATG8 family proteins are a hallmark of many autophagic pathways (6). Although there is only one Atg8 protein in *Saccharomyces cerevisiae*, higher eukaryotes have several ATG8 proteins (subdivided in the LC3 and the GABARAP groups) (7). Atg8 localizes to the isolation membrane and the completed autophagosome and is delivered to the lytic compartment (4, 8). It is attached to the membrane lipid phosphatidylethanolamine by an ubiquitin-like conjugation reaction (9).

The binding of cargo receptors to ATG8 family proteins is conferred by so-called LC3-interacting regions (LIRs)^2^ (10). The core of the LIR motifs are peptides of four amino acids. In a canonical LIR motif of an Atg8-interactor, an aromatic amino acid at position 1 is separated by two varying amino acids from a hydrophobic amino acid at position 4 (^1^W/F/Y-*XX*-^4^L/I/V) (11–15). Upon Atg8 binding, the aromatic and hydrophobic residues are inserted into two hydrophobic pockets of Atg8. Atg8 consists of an ubiquitin-like fold (Ubl) with two additional N-terminal α helices. The first hydrophobic pocket of Atg8 typically accommodates the aromatic residue at position 1 of canonical LIR motifs and is formed by α2 and β sheet 2 of the Ubl fold (12). It is also referred to as the W site because initial studies investigated the structural basis of receptor-ATG8 family protein binding with the LIR motifs of Atg19 (^412^WEEL^415^) and p62 (^338^WTHL^341^), which have a tryptophan at position 1 (11–13). Also, phenylalanine or tyrosine residues in various cargo receptors can bind into this pocket (1, 10, 14). The second hydrophobic pocket, the so-called L site, is formed in the Ubl domain by β2 and α3. The hydrophobic residue at position 4 of the canonical LIR motif inserts into this pocket. In addition to these hydrophobic interactions, LIR motifs form an intermolecular β sheet with ATG8 family proteins (12). Further interactions are mediated by residues outside the four-amino acid core of the LIR motif (11–14).

In *S. cerevisiae*, the autophagy-like cytoplasm-to-vacuole targeting (Cvt) pathway mediates the delivery of the Ape1 (16), Ape4 (17), and Ams1 (18) hydrolases into the vacuole, where they fulfill their enzymatic function. Atg19 serves as cargo receptor in the Cvt pathway and directly interacts with the cargo and Atg8 on the isolation membrane (19–21). The major cargo of the Cvt pathway is Ape1, which is synthesized in the cytoplasm as zymogen (prApe1) with an N-terminal propeptide (22) and is tightly bound by Atg19 (19). The Cvt pathway is a constitutive process and occurs in the absence of other autophagic stimuli. The membranes of Cvt vesicles are closely aligned to the prApe1 cargo (3, 23, 24). As a result, other non-cargo cytoplasmic material is excluded from these vesicles. Upon induction of autophagy by starvation, the prApe1 cargo is also tethered to the autophagosomal membrane in an Atg19-dependent manner, but other cytoplasmic material is not excluded from the larger autophagosomes (3).

It was shown previously that Atg19 harbors multiple Atg8 binding sites in its C-terminal domain and that multiple Atg8 binding sites are required for the Cvt pathway but not for Ape1 transport via autophagosomes (24). One of these Atg8 binding sites is the canonical C-terminal LIR motif (^412^WEEL^415^) (12). The mutation of residues in two further LIR-like sequences, ^376^FYSF^379^ and ^384^LPEL^387^, also affected Atg8 binding, and simultaneous mutation of the canonical and the two LIR-like motifs rendered the interaction of the Atg19 C terminus with Atg8 undetectable (24). However, it was unclear whether the ^376^FYSF^379^ and ^384^LPEL^387^ motifs interact directly with Atg8 and, if so, whether they bind to the same sites in Atg8 as the canonical LIR ^412^WEEL^415^ motif.

Here we show, using NMR spectroscopy, that peptides containing the ^376^FYSF^379^ and ^384^LPEL^387^ motifs interact directly with regions of Atg8 that are overlapping with the binding site for the canonical ^412^WEEL^415^ motif. The affinity of the two non-canonical motifs for Atg8 is lower than that of the canonical LIR motif and, hence, we term them accessory LIR motifs. In a microscopy-based protein-protein interaction assay, we further show that these accessory LIR motifs contribute to the interaction of the Atg19 C terminus with Atg8. We also demonstrate that the integrity of these accessory LIRs is important for the Cvt pathway in *S. cerevisiae*. We propose that multiple low-affinity interactions mediate strong binding of Atg19 to clustered Atg8 and, thus, specific interaction of Atg19 with the isolation membrane on which Atg8 is concentrated.

## Results

**FIGURE 1.**
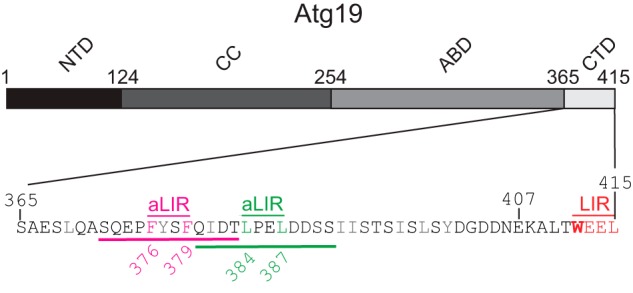
**The C-terminal domain of Atg19.** The Atg19 C terminus (*CTD*, amino acids 365–415) contains several Atg8 binding regions. The canonical LIR motif at the extreme C terminus is labeled in *red. Pink* and *green* labels indicate two putative accessory LIR-like motifs: ^376^FYSF^379^ and ^384^LPEL^387^. The 12 amino acid sequences of synthetic peptides used in this study are *underlined*. Hydrophobic amino acids are shown in *gray. NTD*, N-terminal domain; *CC*, coiled-coil domain (prApe1 cargo interaction); *ABD*, Ams1 binding domain (Ams1 cargo interaction).

**FIGURE 2.**
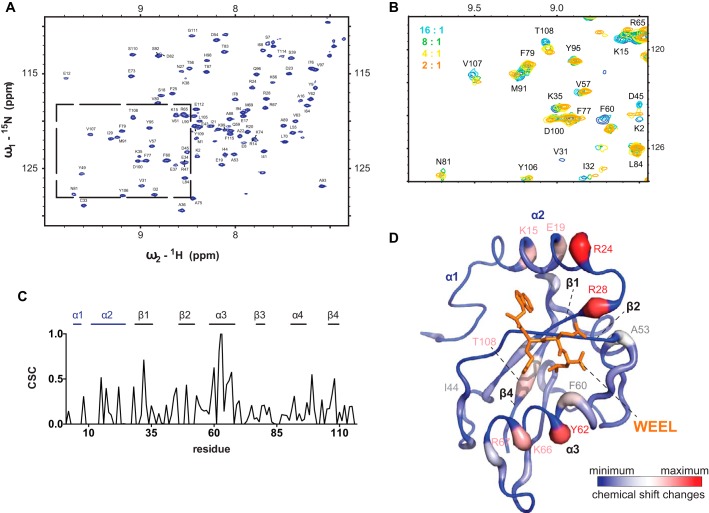
**The atomic environment in Atg8 changes upon titration of the Atg19 CTD.** *A* and *B*, HSQC spectrum of [^15^N]Atg8 alone (*A*) and with increasing concentration of the Atg19 CTD (*B*). ^15^N HSQC spectra of titration steps are color-coded from *blue* to *orange* according to their molar ratios of [^15^N]Atg8:Atg19 C terminus as indicated. *C*, the values of CSCs in parts per million at 1:2 Atg19 CTD to Atg8 were plotted against the residue position in [^15^N]Atg8 (missing values are assigned as CSCs of 0 ppm). Corresponding secondary structure elements in Atg8 are indicated at the *top. D*, the CSCs induced at the 2:1 (Atg8/Atg19 CTD) molar ratio titration point were mapped on the crystal structure of the Atg8-Atg19 LIR motif ^412^WEEL^415^ complex (PDB code 2ZPN (12), the LIR motif is shown as a *stick model* in *orange*). *Blue* indicates no CSC, *red* and *thickened areas* of the peptide chain indicate maximal CSC normalized relative to the maximum experimental CSC of wild-type Atg19 C terminus (0.81 ppm). Secondary structure elements are labeled (*blue font*, ATG8 family-specific, *black font*, ubiquitin-like fold). Representative residues are labeled.

To interrogate the interaction of the C-terminal domain (CTD) of Atg19 with Atg8, we employed two-dimensional ^15^N HSQC by titrating the C-terminal domain of Atg19 (amino acids 365–415, Fig. 1) into ^15^N isotope-labeled Atg8. In particular, we used the K26P mutant of Atg8 (hereafter called Atg8), which is a functionally uncompromised mutant of Atg8 used previously to ensure stability of the protein during the course of the NMR experiment (25). The unlabeled C-terminal domain of Atg19 was added in increasing amounts to Atg8 in molar ratios of Atg19 CTD to Atg8 from 1:16 up to 4:1. These titration ratios were chosen based on the previous observation that one Atg19 C terminus can interact with up to four molecules of Atg8 in size exclusion chromatography (24). We recorded the CSCs in Atg8 upon addition of the wild-type Atg19 C terminus (Fig. 2, *A* and *B*). The sequential assignment of the peaks to the Atg8 structure was taken from (25). Titration of the wild-type Atg19 C terminus resulted in CSCs that were most pronounced for amino acids in Atg8 family-specific helix α2 (Phe^11^-Arg^24^) and helix α3 (Val^57^-Ile^68^) of the ubiquitin-like fold (Fig. 2, *C* and *D*). These regions contribute to the Atg19 interaction by framing the two hydrophobic W and L pockets in Atg8 (12). In α3 of Atg8, the largest shift changes were observed around Phe^60^ to Arg^67^, with Tyr^62^ being most affected (Fig. 2, *C* and *D*). Phe^60^ and Val^63^ may help to stabilize the hydrophobic core of the L site, whereas Arg^67^ has been shown to contact the Glu^413^ residue at position 2 of the canonical LIR motif of Atg19 (12). Arg^28^ also showed CSC upon Atg19 CTD titration. This residue is involved in binding to Glu^414^ at position 3 of the canonical ^412^WEEL^415^ LIR motif of Atg19 (12). Ala^53^ and Asp^54^ are positioned in a loop connecting β2 and α3. The observed CSC in this region may be due to structural rearrangements when a LIR motif binds around β2 and α3. Also, residues between β1 and β2 often showed moderate CSCs (around Glu^37^ to Asp^45^). β2 plays a role in receptor interaction by forming an intermolecular β-sheet with the LIR motif (12). The shift changes in the reverse turn connecting β1 and β2 may hint to conformational rearrangement of this strand upon LIR motif binding. The positions of the CSCs in Atg8 upon addition of Atg19 correspond well with the binding mode of the ^412^WEEL^415^ LIR peptide to Atg8, as determined by x-ray crystallography (12).

**FIGURE 3.**
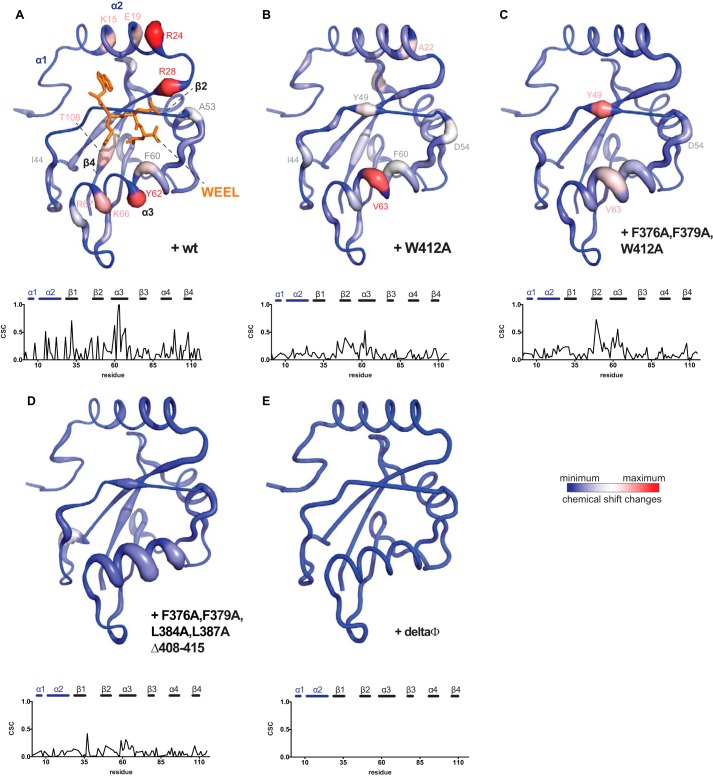
**Comparison of CSCs in Atg8 induced by wild-type and mutant Atg19 CTDs.** *A*, CSCs induced by wild-type Atg19 CTD at a 2:1 (Atg8/Atg19 CTD) ratio mapped onto [^15^N]Atg8 (same as Fig. 2*D*). Corresponding CSCs (parts per million) *versus* residue number plots are shown below the structures. *B–D*, titration of Atg19 W412A CTD into Atg8 induced chemical shift perturbations in similar regions of Atg8 as the Atg19 wild-type CTD (*B*) as well as the Atg19 CTD F376A, F379A, and W412A (*C*) and the Atg19 CTD (amino acids 365–407) F376A, F379A, L384A, and L387A (*D*). *E*, the Atg19 (amino acids 365–407) polyalanine mutant CTD with all hydrophobic residues exchanged to alanines (ΔΦ) induced no CSCs. The color code is as in Fig. 2. Data are normalized to a maximum CSC of 0.81 ppm.

We then went on to determine the effect of mutations in the canonical ^412^WEEL^415^ LIR on the extent and position of the CSCs in Atg8. To this end, Trp^412^ was mutated to Ala, and the mutant Atg19 CTD was titrated into labeled Atg8. Consistent with previous data showing that the ^412^WEEL^415^ is not essential for Atg8 interaction (24), addition of this mutant CTD still resulted in apparent CSCs (Fig. 3, *A* and *B*). Interestingly, the shifts observed for the W412A mutant were in similar positions compared with the wild-type CTD, although Tyr^49^ of β2 (Lys^48^-Pro^52^) was more affected by the W412A mutant Atg19 CTD. This residue is located in the L site and thus may be contacted by the amino acid at position 4 of a LIR motif. It has previously been shown to be involved in receptor binding and autophagic function (26).

The Atg19 C terminus contains two additional LIR-like motifs, ^376^FYSF^379^ and ^384^LPEL^387^ (Fig. 1). Mutations in these LIR-like motifs were shown to result in weaker Atg8-Atg19 interactions (24). To determine whether these motifs are responsible for the CSCs observed in Atg8 upon addition of the W412A mutant, we introduced Phe^376^- and Phe^379^-to-Ala mutations into the W412A C terminus. The overall CSCs in Atg8, upon addition of this triple mutant C terminus, were generally weaker compared with the CSCs caused by the W412A mutant but were still detectable (Fig. 3*C*). Notably, the CSCs were in similar positions compared with the shifts caused by the wild-type and W412A CTDs (Fig. 3, *A* and *B*). We therefore mutated Leu^384^ and Leu^387^ in the second LIR-like motif (Fig. 1) in addition to F376A and F379A mutations. We also deleted the very C-terminal eight amino acids containing the canonical LIR motif of Atg19 (Fig. 1). Introduction of these mutations into the Atg19 C terminus further weakened the CSCs in Atg8 but, surprisingly, still did not completely abolish all CSCs (Fig. 3*D*). Only simultaneous mutation of all hydrophobic amino acids (ΔΦ; Leu^369^, Phe^376^, Tyr^377^, Phe^379^, Ile^381^, Leu^384^, Leu^387^, Ile^392^, Ile^393^, Ile^397^, Leu^399^, and Y400A) and deletion of the last eight amino acids (Fig. 1) in the Atg19 CTD resulted in non-detectable interaction in our NMR experiments (Fig. 3*E*).

**FIGURE 4.**
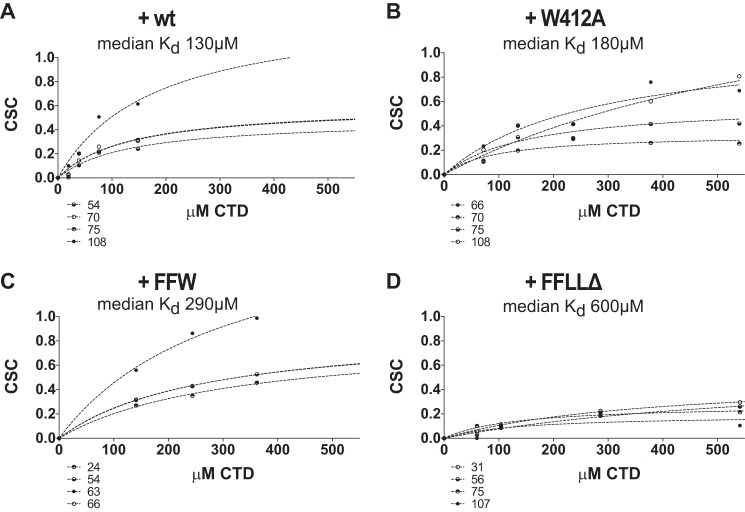
**Estimation of binding affinities from titration-induced CSC.** CSCs at indicated concentrations of representative Atg8 residues (as indicated below) with median estimated dissociation constants of wild-type Atg19 CTD titration (*K_d_* = 130 μm) (*A*); Atg19 W_412_A CTD (*K_d_* = 180 μm) (*B*); Atg19 C terminus Phe^376^, Phe^379^, Leu^384^, Leu^387^, and W412A (*K_d_* = 290 μm) (*C*); and Atg19 C terminus (amino acids 365–407) F376A, F379A, L384A, and L387A (*K_d_* = 600 μm) (*D*). *Dotted lines* represent one-site binding curves modeled with the GraphPad Prism software.

**FIGURE 5.**
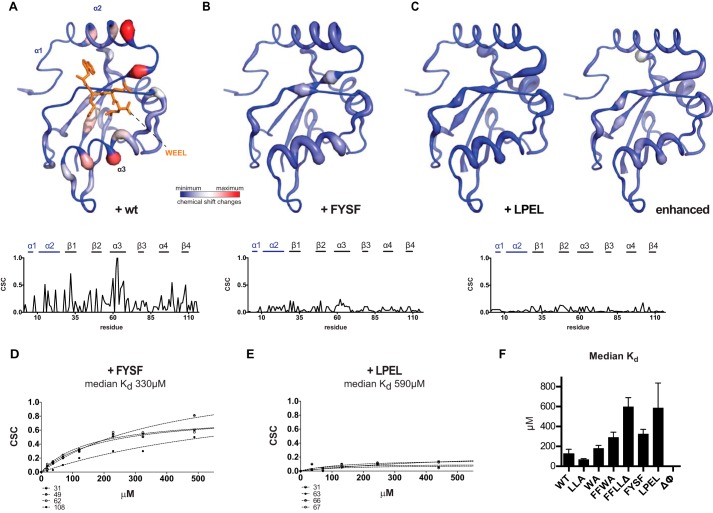
**Accessory LIR motifs of Atg19 induce CSC in overlapping regions of Atg8.** *A*, CSCs induced by wild-type Atg19 CTD at a 2:1 (Atg8/Atg19 CTD) ratio mapped on [^15^N]Atg8 (same as in Fig. 2*D*). Shown is a corresponding CSC *versus* residue plot in the *bottom panel* (as in Fig. 2). *B* and *C*, CSC upon titration of 12-amino acid accessory LIR peptides comprising ^376^FYSF^379^ (*B*) or ^384^LPEL^387^ (*C*, see Fig. 1) induced CSCs in similar regions of Atg8 as the wild-type Atg19 CTD. Data are normalized to a CSC maximum of 0.81 (*left panel*) or 0.4 (*right panel*, enhanced) to highlight the areas of CSCs (*C*, *left panel*). *D*, CSCs of representative Atg8 residues (as indicated below) upon titration of the FYSF peptide with an estimated median *K_d_* of 330 μm. *E*, CSCs of representative Atg8 residues (as indicated below) upon titration of the LPEL peptide with an estimated median *K_d_* of 590 μm. *F*, graph summarizing the median dissociation constants of the NMR titrations in this study. *LLA*, L384A, L387A; *WA*, W412A; *FFWA*, F376A, F379A, W412A; *FFLL*Δ, F376A, F379A, L384A, L387A Δ amino acids 408–415; *FYSF*, 12 amino acid peptide containing ^376^FYSF^379^ (see Fig. 1); *LPEL*, 12 amino acid peptide containing ^384^LPEL^387^ (see Fig. 1); ΔΦ, L369A, F376A, Y377A, F379A, I381A, L384A, L387A, I392A, I393A, I397A, L399A, Y400A.

To derive affinities and *K_d_* values for the Atg8-Atg19 interaction, the CSCs of Atg8 resulting from addition of Atg19 CTDs were analyzed by fitting the concentration dependence to a saturation curve employing a simplified Michaelis-Menten model. Errors in the calculation of *K_d_* arise from limited signal-to-noise ratio of ^15^N HSQC cross-peaks, their finite line width, their scalar ^3^J_HNH_α coupling, and partial peak overlap. Still, the dominant contribution to the error is the residue-to-residue variation of the *K_d_* values extracted from individual fits. We used the median *K_d_* from evaluation of four to twelve amino acids with detectable CSC as an estimate. The estimated *K_d_* of the wild-type Atg19 C terminus was about 130 μm (Fig. 4*A*), which increased to 180 μm upon introduction of the W412A mutation (Fig. 4*B*) and was about 70 μm for the L387/384A mutant (Fig. 5*F*). Additional mutation of F376A, 379A resulted in a further increase to 290 μm (Fig. 4*C*), and silencing the ^384^LPEL^387^ in addition to the canonical ^412^WEEL^415^ LIR and the ^376^FYSF^379^ LIR-like motif resulted in a *K_d_* of 600 μm (Fig. 4*D*).

The finding that mutation of the ^376^FYSF^379^ and ^384^LPEL^387^ LIR-like motifs resulted in an increased *K_d_* and an overall lower magnitude of CSCs (Figs. 2 and 3) suggested that these residues may be in direct contact with Atg8. In addition, the position of the shifts in Atg8 upon mutation of the ^412^WEEL^415^ and ^376^FYSF^379^ motifs suggested that the two LIR-like motifs interact with Atg8 in a manner similar to the canonical ^412^WEEL^415^ motif. To test this hypothesis, we titrated 12 amino acid-long peptides containing the respective LIR-like motif in the center (Fig. 1) into labeled Atg8 and recorded the CSCs (Fig. 5). The peptide containing the ^376^FYSF^379^ motif caused generally smaller CSCs compared with the wild-type Atg19 C terminus (Fig. 5, *A* and *B*). CSCs were also detected in α2 (Phe^11^-Arg^24^) of Atg8, indicating that the Phe residues may interact with the W site in Atg8 (Fig. 2, *C* and *D*). Titration of the ^384^LPEL^387^ peptide caused even smaller CSCs in Atg8, but CSCs were still readily detectable (Fig. 5*C*). Notably, both peptides caused CSCs in α3 and, thus, in positions similar to the position occupied by the canonical LIR motif (12), suggesting that their mode of binding is very similar (Fig. 5, *A–C*). Using the magnitude of the CSCs in dependence of the Atg19 concentration, we estimated the *K_d_* values of ^376^FYSF^379^ and ^384^LPEL^387^ to be around 330 μm and 590 μm, respectively. Fig. 5*F* shows a summary of the estimated *K_d_* values of the measurements described above. We attempted to determine a more exact *K_d_* for the FYSF and LPEL peptides by isothermal titration calorimetry. However, although we could determine a *K_d_* of 2.8 μm for a peptide containing the canonical ^412^WEEL^415^ motif (Atg19, ^403^GDDNEKALTWEEL^415^), we did not obtain any interpretable ITC measurements for the two FYSF and LPEL peptides, likely because of their low affinity (Fig. 5, *D–F*).

**FIGURE 6.**
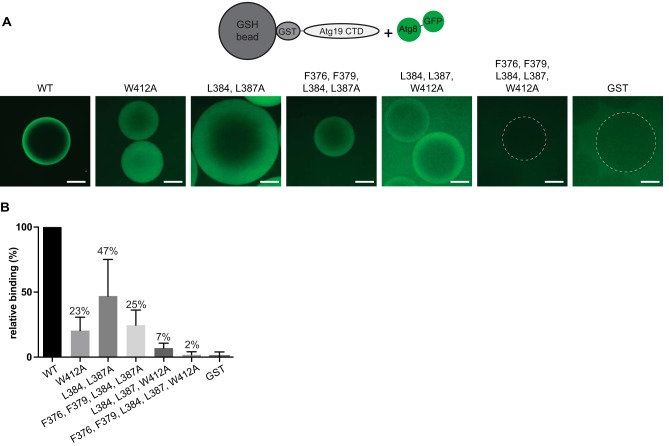
**Accessory LIR motifs contribute to Atg8 binding in a microscopy-based interaction assay.** *A*, glutathione-Sepharose beads were coated with GST-tagged Atg19 CTDs, washed, incubated in a 1 μm EGFP-Atg8 solution, and imaged by spinning disc microscopy. *B*, signal intensity of EGFP-Atg8 decreased for the LIR and accessory LIR mutants. *C*, quantification of the EGFP signal (mean ± S.D. from 3 experiments) on GST-Atg19 CTD-coated beads normalized to the mean signal on wild-type GST-Atg19 CTD-coated beads. *Scale bars* = 30 μm.

To confirm the contributions of the ^376^FYSF^379^ and ^384^LPEL^387^ motifs for Atg8 binding, we employed a microscopy-based protein-protein interaction assay. To this end, EGFP-Atg8 in solution was recruited to glutathione-Sepharose beads coated with GST-Atg19 C termini (Fig. 6, *A* and *B*). Atg8 bound efficiently to beads coated with the wild-type C terminus. Upon introduction of the W412A mutation, the Atg8 signal on the beads dropped to 23%. Consistent with a contribution of the ^384^LPEL^387^ motif to the interaction with Atg8, the L384A, L387A mutant showed a reduction of Atg8 signal to 47%. Additional mutation of Phe^376^ and Phe^379^ to Ala reduced the Atg8 signal to 25%, whereas simultaneous mutation of Phe^376^, Phe^379^, Leu^384^, Leu^387^, and Trp^412^ to Ala rendered the Atg8 recruitment to the beads undetectable above the GST background control (Fig. 6*B*).

**FIGURE 7.**
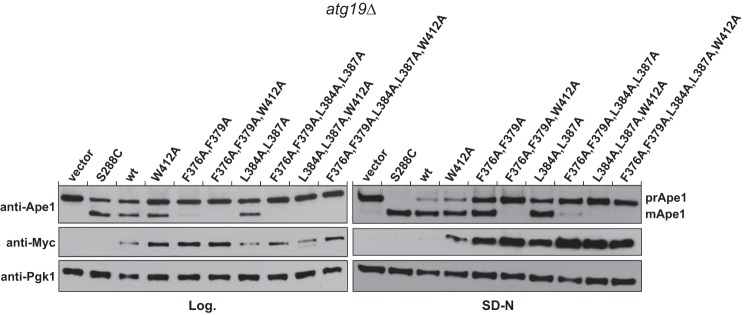
**Atg19 LIR mutants are impaired in prApe1 maturation.** Vacuolar processing of prApe1 was monitored in Δ*atg19* cells in logarithmic growth phase (*Log.*) and under nitrogen starvation conditions (*SD-N*). Shown are Western blots using the indicated antibodies.

Next we determined the effect of mutations in the LIR-like motifs of Atg19 for prApe1 processing during the Cvt pathway (Fig. 7, *Log.*) and autophagy (Fig. 7, *SD-N*). To this end, we expressed various mutant forms of Atg19 as Myc-tagged proteins in Atg19-deficient cells. As controls, we transformed these cells with a plasmid containing Atg19 without any mutations, and the wild-type *S. cerevisiae* (S288C) strain was transformed with an empty plasmid. Consistent with earlier findings, we found that mutation of Trp^412^ in the canonical LIR motif had a mild effect on Ape1 processing both during the Cvt pathway and autophagy. Simultaneous mutation of Phe^376^ and Phe^379^ to Ala had a severe effect on prApe1 processing during the Cvt pathway but not in autophagy. This effect was more pronounced than reported previously (24), likely because of the fact that the cells were grown to a lower cell density in this study. Additional mutation of Trp^412^ to Ala completely abolished prApe1 processing by the Cvt pathway and rendered it almost undetectable during autophagy. Introduction of the L384A/L387A double mutation had no major effect on prApe1 processing. However, when combined with F376A, F379A, or W412A mutations, it became apparent that these residues contribute to prApe1 transport into the vacuole. The Phe^376^-, Phe^379^-, Leu^384^-, Leu^387^-, and Trp^412^-to-Ala mutation resulted in a completely non-functional Atg19 protein both under Cvt pathway and autophagy conditions.

## Discussion

Here we have shown that the Atg19 cargo receptor carries multiple LIR motifs in its C-terminal domain that directly interact with the same sites in Atg8. As a result, one Atg19 molecule can interact with multiple Atg8 molecules simultaneously, conferring specific binding of Atg19 to the isolation membrane, where Atg8 is concentrated.

It was shown previously that Atg19 contains multiple interaction sites in its C-terminal domain (24), but their mode of interaction with Atg8 was unclear. Here we have employed NMR, using the Atg19 C terminus titrated into ^15^N-labeled Atg8, to monitor which amino acids change their chemical environment upon Atg19 binding, as determined by two-dimensional ^15^N HSQCs. The main regions affected were residues in the Atg8 family-specific α2 (Phe^11^-Arg^24^) and in the Ubl fold at α3 (Val^57^-Ile^68^). These regions frame the W and L sites, respectively, of the canonical LIR-binding regions (12, 13). We did not observe any major shifts in residues that are remote from these regions, indicating that all regions in Atg19 that interact with Atg8 bind to the same sites. In fact, mutational analysis and the titration of peptides containing two LIR-like motifs, ^376^FYSF^379^ and ^384^LPEL^387^, strongly suggest that these motifs directly interact with Atg8 in a manner analogous to the canonical ^412^WEEL^415^ LIR motif. The estimated *K_d_* values of their interaction are 330 μm and 590 μm, respectively, and thus very low. The fact that our NMR-based estimate of 130 μm for the *K_d_* of the wild-type CTD-Atg8 interaction is lower than the ITC-based *K_d_* determination with the canonical LIR peptide (2.8 μm) and full-length Atg19 (35 μm (24)) may be due to fact that this estimate is a convolution of multiple high- and low-affinity interactions. The *K_d_* of the low-affinity binding site mutant L384A, L387A (70 μm) is actually closer to these values than the wild-type CTD. This variation is due to the limited precision of NMR-based affinity determination in this concentration range. It may also reflect the fact that the observed overall *K_d_* is less influenced by the mutation of lower-affinity peptide motifs as the high affinity ^412^WEEL^415^ LIR motif will dominate the interaction. *In vitro* pulldown assays (Fig. 6) of the L384A, L387A mutant showed a decreased interaction with Atg8. This is likely due to the fact that the number of Atg8 proteins that can be bound by the L384A, L387A mutant C terminus is lower than that of the wild-type protein.

We term the additional LIR-like sequences accessory LIR motifs because it is unlikely that they mediate relevant interactions with Atg8 in isolation. However, when they are brought in proximity to Atg8 concentrated on the membrane by canonical LIR motifs, it is likely that they contribute to avid binding of the Atg19 receptor to isolation membranes. Given the close proximity of the two accessory LIR motifs (Fig. 1), it is unclear whether they can interact with two Atg8 molecules simultaneously. Perhaps the spacer between the two LIR motifs forms a hairpin, and one of these LIR motifs interacts with Atg8 in an antiparallel manner. Surprisingly, in our NMR experiments, the interaction between the Atg19 C terminus and Atg8 was fully abrogated only upon mutation of all hydrophobic amino acids in the C terminus. This suggests that even single hydrophobic amino acids can measurably interact with the W and L sites of Atg8, at least at the high protein concentration used for the NMR experiments. Presumably, these interactions also occur *in vivo* when the Atg19 cargo receptor is sandwiched between the prApe1 cargo and the Atg8-coated isolation membrane.

To corroborate our NMR-based interactions studies, we attempted to measure their interaction with Atg8 by isothermal titration calorimetry. However, we could not obtain any measurable signal above background upon titration of peptides containing the ^376^FYSF^379^ or ^384^LPEL^387^ motif. We therefore employed a microscopy-based protein-protein interaction by coupling the Atg19 C terminus to glutathione beads via GST. We then added recombinant EGFP-Atg8 and determined its bead-associated signal by spinning disc microscopy. The advantage of this assay over, for example, classical pulldown assays is that it does not require washing steps, and it is thus able to detect interactions with high off rates. This assay revealed a contribution of the ^376^FYSF^379^ or ^384^LPEL^387^ motifs to the Atg8-Atg19 interaction. To determine the relevance of the two accessory LIR motifs for prApe1 processing *in vivo*, we determined its maturation during the Cvt pathway and nitrogen starvation-induced autophagy. We have previously reported the importance of the ^376^FYSF^379^ motif for prApe1 processing by analyzing the effect of the F376A, F379A mutation. Here we have shown that the L384A, L387A mutation also reduces prApe1 processing. This is not apparent when these residues are mutated in the absence of additional LIR-disrupting mutations but becomes detectable when combined with the F376A, F379A, or W412A mutations. In contrast to our previous study (24), the F376A, F379A mutation affected prApe1 processing during the Cvt pathway more severely. This may be due to the fact that we analyzed the prApe1 processing at a lower cell density in this study. The drastic effect of the F376A, F379A mutation points to additional functions of this motif specifically during the Cvt pathway.

**FIGURE 8.**
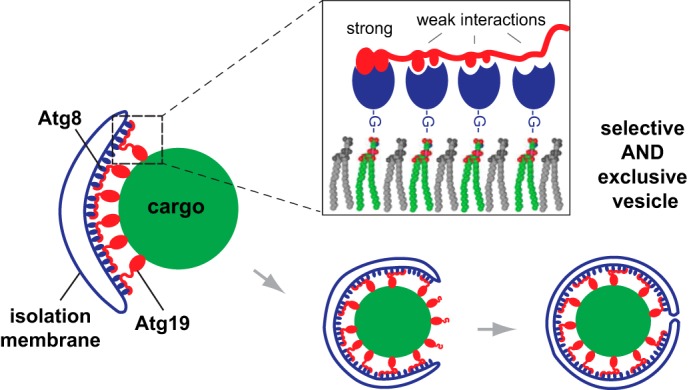
**Atg19 binds Atg8 via several sites in its C terminus.** Atg19 contains a canonical LIR motif and two low-affinity accessory LIR motifs in its C-terminal domain to bind to Atg8, which decorates the isolation membrane. Nonspecific hydrophobic interactions may further stabilize the binding. These multiple Atg8 binding sites mediate a high-avidity interaction and enable the receptor Atg19 to closely appose the autophagy cargo and the growing isolation membrane. Unwanted cytoplasmic material is therefore excluded from the Cvt vesicle.

Taken together, our data suggest that Atg19 interacts with Atg8 via three sites, one canonical LIR motif and two accessory LIR motifs. Each of these motifs, and in particular the accessory LIR motifs, have a low affinity for Atg8. However, in combination, they confer a high-avidity interaction with clustered Atg8 and therefore select for Atg8 proteins when they are concentrated on isolation membranes as opposed to soluble Atg8 in the cytosol. During the Cvt pathway, this high-avidity interaction mediates the close apposition of the isolation membrane and the cargo, excluding non-cargo material from delivery into the vacuole (Fig. 8). Other cargo receptors may use a similar mechanism or oligomerization during selective autophagy (1, 27).

## Experimental Procedures

### 

#### 

##### Protein Expression and Purification

Open reading frames (*S. cerevisiae* Atg19 amino acids 365–415 or 407, Atg8 1–117) were cloned into the pGEX-4-T1 vector (GE Healthcare) via 5′ EcoRI and 3′ SalI or 5′ BamHI and 3′SalI restriction sites (all enzymes were from New England Biolabs). Point mutations were introduced via site-directed mutagenesis using complementary primers containing the desired nucleotide exchange (see supplemental Table 1 for primer sequences).

Proteins were expressed in *Escherichia coli* Rosetta pLysS DE3 (Merck Millipore). Cells were grown in lysogeny broth (Roth) at 37 °C, and expression was induced at 18 °C at an optical density of 0.8 with 0.1 mm isopropyl-β-d-thiogalactopyranoside (Discovery Fine Chemicals). Atg8 WT and K26P were ^15^N isotope-labeled by expression in M9 minimal medium containing [^15^N]ammonium chloride (Cambridge Isotope Laboratories).

Cells were harvested by centrifugation and resuspended in 50 mm HEPES (pH 7.5) (AppliChem), 300 mm NaCl (VWR Chemicals), 1 mm MgCl_2_ (Sigma-Aldrich), DNase (Sigma-Aldrich), 1 mm Pefabloc (Sigma-Aldrich), and 2 mm β-mercaptoethanol (Merck). Cell were lysed by freeze-thawing and sonication. The lysate was cleared by centrifugation (142,000 × *g* (35,000 rpm, Beckman Ti45 rotor) for 45 min at 4 °C). The protein in the supernatant was bound for 1 h at 4 °C to glutathione 4B-Sepharose beads (GE Healthcare). After five washes with 50 mm HEPES (pH 7.5), 300 mm NaCl, and 2 mm β-mercaptoethanol of 10 min each and one wash with 50 mm HEPES (pH 7.5), 700 mm NaCl, and 2 mm β-mercaptoethanol for 30 min, thrombin protease (Serva) was added to cleave the protein off the GST tag overnight at 4 °C. The cleaved protein in the supernatant was filtered and concentrated in 3 kDa molecular weight cutoff or 10 kDa molecular weight cutoff concentration tubes (Merck Millipore) to about 2 ml, loaded on a Sepharose 75pg 16/600 size exclusion chromatography column (GE Healthcare), and eluted at a 0.5 ml/min flow rate in 25 mm HEPES (pH 7.5), 150 mm NaCl, and 1 mm DTT (AppliChem) on an Äkta Pure or Prime system. The fractions containing the protein (confirmed by SDS-PAGE and Coomassie staining) were pooled and concentrated to obtain a 0.3–3 mm protein concentration. Protein concentrations were estimated from the absorption at 280- or 205-nm wavelength. Synthetic peptides were purchased from Eurogentec.

##### NMR

Two-dimensional ^15^N HSQC spectra were acquired at ambient temperature (25 °C) on Varian Inova 500, Direct Drive 600, or Bruker Avance 3 HD+ 600 spectrometers operating at 500–600 MHz (^1^H frequency). Gradient enhancement using triple-resonance (H/N/C) z axis pulsed field gradient probes was applied. Typical protein concentrations of Atg8 ranged between 135–500 μm and of Atg19 C terminus between 15–1000 μm.

##### Dissociation Constant Estimation

Dissociation constants of each Atg19 C terminus mutant and Atg8 were estimated by relating the Atg19 C terminus concentration to the chemical shift changes (CSCs) the respective mutant caused in Atg8, assuming a Michaelis-Menten model. Composite chemical shift changes were calculated as follows: CSC = sqrt{Δσ(^15^N)^2^+[5Δσ(^1^H)]^2^}. Between the two extremes of free Atg8 and fully saturated Atg8, the concentration that results in a half-maximum CSC, *i.e.* where half of the molecules are bound, corresponds to the dissociation constant *K_d_*. Relating to the CSC *versus* concentration of Atg19 C terminus plot, this would provide an estimate of the dissociation constant *K_d_* of Atg19 C terminus to Atg8 where half of the molecules are bound to Atg8. Nevertheless, the *K_d_* values were more precisely determined by fitting the concentration dependence of the CSC to a saturation curve of the form CSC([Atg19]) = CSCmax*([Atg19])/{([Atg19])+*K_d_*}. Atg8 amino acids with a CSC significant enough for analysis were selected for each experiment (four to twelve amino acids for each Atg19 C terminus titration experiment).

##### GST Affinity Pulldowns

Glutathione-Sepharose 4B beads (GE Healthcare) were incubated with GST-tagged Atg19 C terminus for 1 h at 4 °C. After washing the beads three times with 25 mm HEPES (pH 7.5), 150 mm NaCl, and 1 mm DTT, 1 μl of a 1:1 resuspension of glutathione beads in protein solution was added to a 1 μm solution of EGFP-Atg8 30 min prior to imaging. Microscopic images were acquired using a confocal spinning disc microscope (Visitron) and processed with ImageJ software.

##### PrApe1 Processing

Δ*atg19* cells in stationary phase grown on non-selective yeast extract peptone plates with 2% glucose were transformed with pRS316–6xmyc-Atg19 (WT or LIR mutant versions) and grown on selection plates (synthetic defined medium containing 2% glucose, 1.7 g/liter yeast nitrogen base without amino acids and ammonium sulfate (ForMedium), 5 g/liter ammonium sulfate, 20 g/liter glucose, and 25 g/liter agarose, supplemented with amino acids (complete supplement mixture, ForMedium)). One to four colonies of stationary phase cells were regrown to an *A*_600_ > 1 in synthetic defined medium without uracil, subsequently diluted in SD-complete, and grown to *A*_600_ = 1. 1.4 ml of cells was broken in 14% trichloroacetic acid, washed with acetone, and dissolved in 35 μl per 1 unit of optical density (*A*_600_) 116 mm Tris (pH 6.8), 4.9% glycerol, 8 m urea, 143 mm mercaptoethanol, and 8% bromophenol blue. 8 μl was separated on 10% bis-acrylamide SDS gels. Ape1 was detected by Western blotting using a rabbit polyclonal anti-Ape1 antibody (1:10,000, a kind gift from Claudine Kraft, University of Vienna).

6xMyc-Atg19 was detected using an anti-Myc tag antibody (clone 4A6, diluted 1:500). Anti-Pgk1 (diluted 1:10,000) was purchased from Invitrogen (catalog no. 459250). A wild-type strain transformed with pRS316 vector served as a positive control.

##### Yeast Strains

The yeast strains used in this study can be found in supplemental Table 1. The genotype of the *S. cerevisiae* S288C genetic background used here is his3Δ1; leu2Δ0; met15Δ0; ura3Δ0. Knockout strains were purchased as diploids from EUROSCARF, and haploid spores were selected.

##### Yeast Expression Constructs

Plasmids for yeast expression were produced as described in Ref. 24. To introduce point mutations in the Atg19 open reading frame, primers as listed in supplemental Table 1 were used.

## Author Contributions

C. A. and S. M. conceived and designed the experiments and wrote the manuscript with comments from G. K. C. A. performed most experiments, apart from G. K., who performed the NMR experiments and analyzed the NMR data. S. M. supervised the experiments.

## Supplementary Material

Supplemental Data

## Footnotes

2 The abbreviations used are:

LIR: LC3-interacting region
Cvt: cytoplasm-to-vacuole targeting
CTD: C-terminal domain
CSC: chemical shift change
HSQC: heteronuclear single quantum correlation
EGFP: enhanced green fluorescent protein.
