# Supplementary material for: Accessory Interaction Motifs in the Atg19 Cargo Receptor Enable Strong Binding to the Clustered Ubiquitin-related Atg8 Protein
Source: J Biol Chem. 2016 Jul 11;291(36):18799–808. doi: 10.1074/jbc.M116.736892 (PMC5009254; doi:10.1074/jbc.M116.736892)
Supplement: Supplemental Data [file supp_291_36_18799__index.html]

Accessory Interaction Motifs in the Atg19 Cargo Receptor Enable Strong Binding to the Clustered Ubiquitin-Related Atg8 Protein — Accessory Interaction Motifs in the Atg19 Cargo Receptor Enable Strong Binding to the Clustered Ubiquitin-related Atg8 Protein — Atg19 Binds Atg8 via Multiple Sites — Supplemental Data 

# Accessory Interaction Motifs in the Atg19 Cargo Receptor Enable Strong Binding to the Clustered Ubiquitin-related Atg8 Protein

## Supplemental Data

- Supplemental Table 1 (.pdf, 79 KB) - This table contains a list of primers and yeast strains.
