# Supplementary material for: Accessory Interaction Motifs in the Atg19 Cargo Receptor Enable Strong Binding to the Clustered Ubiquitin-related Atg8 Protein
Source: J Biol Chem. 2016 Jul 11;291(36):18799–808. doi: 10.1074/jbc.M116.736892 (PMC5009254; doi:10.1074/jbc.M116.736892)
Supplement: Supplemental Data [file 10.1074_M116.736892_jbc.M116.736892-1.pdf]

TABLE S1: Primers and yeast strains used in this study.

**Primers**				
Sequence	ID	Restriction	Orientation	Mutation
CCCCCCCCGAATTCTCTGCAGAAAGTTTACAGGC	SMP328	EcoRI	forward	wild type
CCCCCCCCCGTCGACCTAGAGTTCTTCCCAAGTCAG	SMP320	SalI	reverse	wild type
CCCCCCGTCGACCTATTCATTGTCGTCACCGTCATA	SMP466	SalI	reverse	365-407
CCCCCCCCGGATCCTCTGCAGAAAGTTTACAGGC	SMP1116	BamHI	forward	wild type
GAAAAAGCCCTGACTGCGGAAGAACTCTAGGTC	SMP330		forward	W412A
GACCTAGAGTTCTTCCGCAGTCAGGGCTTTTTC	SMP331		reverse	
AGAGCCATTTTACTCCGCTCAAATCGATACGTTA	SMP761		forward	F376, F379A
TAACGTATCGATTTGAGCGGAGTAAAATGGCTCT	SMP762		reverse	F376, F379A
CCTTTCAAATCGATACGTTAGCAGCACTGGATGACT CTAGTATC	SMP873		forward	P385, E386A
GATACTAGAGTCATCCAGTGCTGCTAACGTATCGAT TTGAAAGG	SMP874		reverse	P385, E386A
GCA CCA GAA GCT GAT GAC TCT AGT ATC ATC AGT AC	SMP1084		forward	L384, L387, F379A
CGT ATC GAT TTG AGC GGA GTA AG	SMP1085		reverse	F379A
CGTATCGATTTGAAAGGAGTAA	SMP1438		reverse	L384, L387A
GCC GCC AGT ACA TCC ATT TCA CTC TCT GCT GA	SMP1109		forward	I392, I393, Y401A
ACT AGA GTC ATC AGC TTC TGG TGC	SMP1110		reverse	I392, I393, Y401A
CCC CCG GAT CCT CTG CAG AAA GTG CAC AGG CAT CCC AAG AGC CAG CTG CCT CCG CTC AAG CCG ATA C	SMP1293		forward	Y377A, I381A
**Yeast strains**				
Strain	ID	Background	Genotype	Source
wild type	yCK566	S288C	MATa	Euroscarf
*atg19*Δ	yCK797	S288C	MATa, *atg19*Δ::kanMX6	Euroscarf
